# Holmium laser enucleation of the prostate with Virtual Basket mode: faster and better control on bleeding

**DOI:** 10.1186/s12894-021-00797-5

**Published:** 2021-02-23

**Authors:** Giorgio Bozzini, Matteo Maltagliati, Umberto Besana, Lorenzo Berti, Albert Calori, Maria Chiara Sighinolfi, Salvatore Micali, Jean Baptiste Roche, Ali Gozen, Alexander Mueller, Dimitry Pushkar, Evangelos Liatsikos, Marco Boldini, Carlo Buizza, Bernardo Rocco

**Affiliations:** 1 Department of Urology, ASST Valle Olona, Via Arnaldo da Brescia, 21052 Busto Arsizio, VA Italy; 2 ESUT, European Section for UroTechnology, Arnhem, Italy; 3 Department of Urology, Ospedale Policlinico e Nuovo Ospedale Civile S. Agostino Estense, University of Modena and Reggio Emilia, Modena, Italy; 4 Department of Urology, Clinique Saint Augustin, Bordeaux, France; 5 Department of Urology, SLK Kliniken, Heilbron, Germany; 6 Department of Urology, Spital Limmattal, Schlieren, Switzerland; 7 Department of Urology, Moscow State University, Moscow, Russia; 8 Department of Urology, University of Patras, Patras, Greece; 9 Department of Urology, Clinica Sant’Anna, Lugano, Switzerland

**Keywords:** Benign prostatic hyperplasia, Holmium laser, Laser therapy, Prostate

## Abstract

**Background:**

To compare clinical intra and early postoperative outcomes between conventional Holmium laser enucleation of the prostate (HoLEP) and Holmium laser enucleation of the prostate using the Virtual Basket tool (VB-HoLEP) to treat benign prostatic hyperplasia (BPH).

**Methods:**

This prospective randomized study enrolled consecutive patients with BPH, who were assigned to undergo either HoLEP (n = 100), or VB-HoLEP (n = 100). All patients were evaluated preoperatively and postoperatively, with particular attention to catheterization time, operative time, blood loss, irrigation volume and hospital stay. We also evaluated the patients at 3 and 6 months after surgery and assessed maximum flow rate (Qmax), postvoid residual urine volume (PVR), the International Prostate Symptom Score (IPSS) and the Quality of Life score (QOLS).

**Results:**

No significant differences in preoperative parameters between patients in each study arm were found. Compared to HoLEP, VB-HoLEP resulted in less hemoglobin decrease (2.54 vs. 1.12 g/dl, *P* = 0.03) and reduced operative time (57.33 ± 29.71 vs. 42.99 ± 18.51 min, *P* = 0.04). HoLEP and VB-HoLEP detrmined similar catheterization time (2.2 vs. 1.9 days, *P* = 0.45), irrigation volume (33.3 vs. 31.7 l, *P* = 0.69), and hospital stay (2.8 vs. 2.7 days, *P* = 0.21). During the 6-month follow-up no significant differences in IPSS, Qmax, PVR, and QOLS were demonstrated.

**Conclusions:**

HoLEP and VB-HoLEP are both efficient and safe procedures for relieving lower urinary tract symptoms. VB-HoLEP was statistically superior to HoLEP in blood loss and operative time. However, procedures did not differ significantly in catheterization time, hospital stay, and irrigation volume. No significant differences were demonstrated in QOLS, IPSS, Qmax and PVR throughout the 6-month follow-up.

*Trial Registration*: Current Controlled Trials ISRCTN72879639; date of registration: June 25th, 2015. Retrospectively registred.

## Background

Benign prostatic hyperplasia (BPH), with consequent lower urinary tract symptoms (LUTS), is one of the most common diseases in aging men. Many surgical treatments are available to handle BPH refractory to pharmacological therapy [1]. Transurethral resection of the prostate (TURP) remains the gold standard surgical treatment for prostates with a total volume between 30 and 80 ml. Open prostatectomy (OP), instead, is used for enlarged glands (> 80 ml) [2]. Today, laser enucleation of the prostate is gradually replacing these old techniques due to the advantage of decreased bleeding complications and increased safety. Laser procedures are indicated for the treatment of prostates > 80 ml and they can be considered as alternatives to TURP for prostates with a total volume between 30 and 80 ml [1, 3].

BPH laser surgery comprises many different technologies and techniques [4]. HoLEP was introduced 20 years ago by Fraundorfer and Gilling [5] and, since then, several studies have demonstrated that it determines a reduction in hospital stay, catheterization time, and intraoperative and postoperative bleeding [6]. During the procedure, the surgeons detach the adenoma from the prostate surgical capsule with a blunt dissection, using the holmium laser and the tip of the resectoscope. The laser also allows to perform an accurate hemostasis. With regard to energy and frequency settings, 1.8–2.5 J and 20–50 Hz are normally used, delivering a total power of 80–120 W.

Thanks to its pulsed activity, the Ho:YAG laser is also ideal for stone lithotripsy. However, the energy impact against the stone can cause its migration from the ureter to the renal cavities, or from one calyx to another. Stone migration increases operative time, patient morbidity and healthcare cost. Antiretropulsion devices have been created to prevent stone migration. The “Virtual Basket™” technology is a result of the laser’s pulse modulation, usually employed for holmium laser lithotripsy: the laser emits part of the energy to create an initial bubble and the remaining energy is discharged once the bubble is formed, so that it can pass through the previously created vapor channel.

We applied the Virtual Basket™ mode to HoLEP (VB-HoLEP), in order to compare clinical, intra and early postoperative outcomes between conventional HoLEP and VB-HoLEP for the treatment of benign prostatic hyperplasia (BPH).

## Methods

This prospective randomized study enrolled consecutive patients with BPH who received an indication to HoLEP according to EAU GuideLines [1]. Ethical committee approval was obtained (No. 2019/267 ATSIns) and a subsequent consent form was signed by each patient that entered the study (Clinical trial registration: ISRCTN72879639). A simple 1:1 randomization was used to assign each patient to either HoLEP, or VB-HoLEP. Exclusion criteria were: age under 18, or over 90, presence of acute infection (fever more than 38°C, total leucocyte count more than 15,000/dl or preoperative positive urinary colture), coexisting urethral, or prostate disease and presence of bladder stones. Furthermore, all the recruited patients who refused to give their consent to the study were excluded. Coagulation during the procedure was performed by only using the laser and not with a monopolar, or bipolar resector.

Both groups were treated using the Cyber Ho 100 laser platform (Quanta System, Samarate, Lombardia, Italy) set to 1.8 J at 45 Hz for cutting, with 550 µm reusable laser fibres. The Virtual Basket mode was enabled on the left pedal (used for cutting) in the second group only. The same settings in both groups were also used for coagulation (0.6 J at 35 Hz). The adopted technique was the traditional 3 lobes technique.

A Storz resectoscope with a 12 degrees optic, a Kuntz element (Karl Storz Tuttlingen Germany) and a guide (to allow the 550 µm fiber to pass) were used for all the procedures.

After completing the enucleation, the dissected tissue was morcellated with the DrillCut morcellator (Karl Storz, Tuttlingen, Germany).

All of the patients were evaluated postoperatively with regards to blood loss, catheterization time, irrigation volume, hospital stay and operative time. At 3 and 6 months after surgery, patients were also evaluated with the International Prostate Symptom Score (IPSS), the Quality of Life Score (QOLS), maximum urinary flow rate (Qmax) and postvoid residual urine volume (PVR).

### Statistical analysis

Simple Block Randomization was obtained using the “Adaptative Randomization” software (University of Texas) to reach a good number balance between the two groups. To reach a good allocation concealment we used a centralized service to rule all the partecipanting centers. To avoid any outcome bias blinding of the participants was ensured for the duration of hospitalization (they actually did not know which surgical technique was used for their enucleation) and data were never analyzed by one of the operating surgeons.

A statistical analysis was carried out to assess patients’ data and outcomes. All of the reported *P*-values were obtained with the two-sided exact method at the conventional 5 % significance level. Data were analyzed with the April 2016 by R software v.3.2.3 (R Foundation for Statistical Computing, Vienna, Austria), according to previously published guidelines for the reporting of statistics. We calculated the sample size with a confidence level of 95% and a confidence interval of 5%.

## Results

**Fig. 1:**
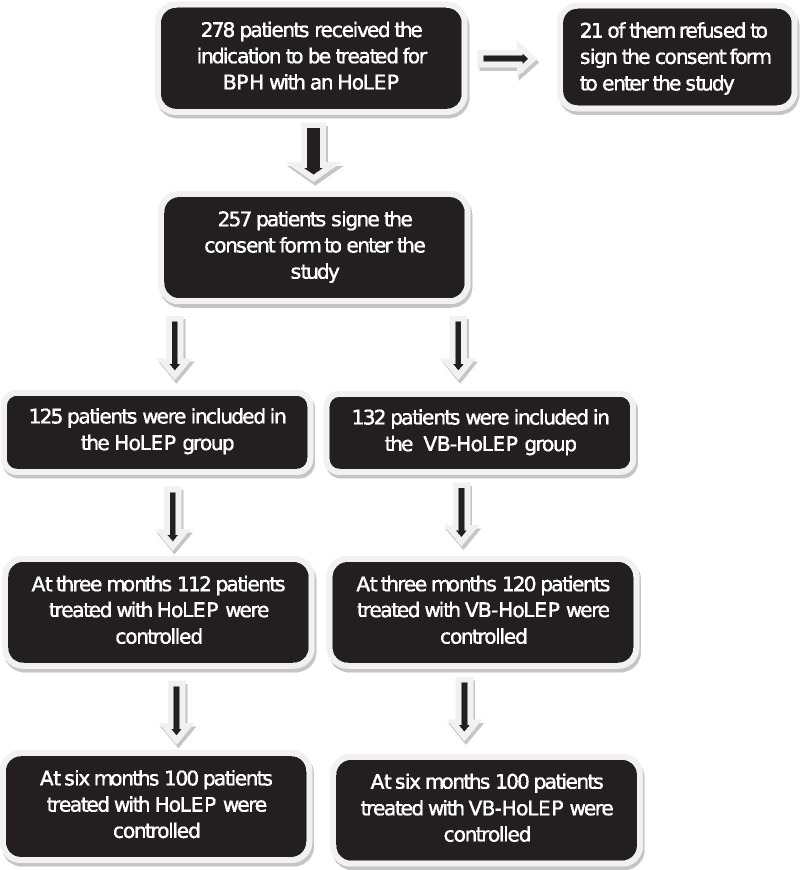
CONSORT flow chart

From June 2019 to January 2020, 278 patients received the indication to be treated with a HoLEP procedure for BPH and met the inclusion criteria of the study. 21 of them refused to sign the consent, leaving 125 patients assigned to the HoLEP group and 132 to the VB-HoLEP one. Three months after surgery 112 and 120 patients were controlled and 100 patients for each arm were able to attend the 6-month scheduled control. CONSORT flow chart Fig. 1.

Patients’ preoperative data are presented in Table 1. Early postoperative outcomes are summarized in Table 2. No significant differences in preoperative parameters between the two study arms were found. Compared to HoLEP, VB-HoLEP resulted in reduced hemoglobin decrease (2.54 vs. 1.12 g/dl, *P* = 0.03) and operative time (57.33 ± 29.71 vs. 42.99 ± 18.51 min, *P* = 0.04). Patients in the HoLEP and VB-HoLEP groups presented similar catheterization time (2.2 vs. 1.9 days, *P* = 0.45), irrigation volume (33.3 vs. 31.7 l, *P* = 0.69), and hospital stay (2.8 vs. 2.7 days, *P* = 0.21). During the 6-month follow-up no significant differences in IPSS, Qmax, PVR and QOLS were found (Table 3).

**Table 1:** Patient’s data

	Group A Holep	Group B VB Holep	*P*
No.	100	100	> 0.05
Age yrs (mean ± SD)	72.1 ± 11.6	70.9 ± 12.8	> 0.05
Preoperative prostatic volume ml. (mean ± SD)	74.2 ± 36.2	77.1 ± 29.4	> 0.05
PSA ng/ml (mean ± SD)	2.7 ± 4.12	2.8 ± 3.89	> 0.05
Preoperative Hb g/dl (mean ± SD)	13.4 ± 2.45	13.9 ± 2.23	> 0.05
IPSS (mean ± SD)	19.9 ± 7.01	18.1 ± 6.69	> 0.05
Q max ml/sec (mean ± SD)	6.9 ± 5.54	7.1 ± 6.12	> 0.05
Post void volume ml (mean ± SD)	118.8 ± 161.95	124.1 ± 148.92	> 0.05

**Table 2:** Intra and early post operative outcomes and postoperative complications

	Group A	Group B	*P*
Operative time, min (mean ± SD)	57.33 ± 29.71	42.99 ± 18.51	= 0.04
Haemoglobin decrease, g/dl (mean ± SD)	2.54 ± 1.23	1.12 ± 1.78	= 0.03
Catheterization time, days (mean ± SD)	2.2 ± 3.55	1.9 ± 2.81	= 0.45
Continuous irrigation volume, liters (mean ± SD)	33.3 ± 24.78	31.7 ± 25.22	= 0.69
Enucleated/resected prostatic volume, g (mean ± SD)	47.75 ± 18.54	51.03 ± 14.84	= 0.321
Hospital stay, days (mean ± SD)	2.8 ± 3.19	2.7 ± 2.89	= 0.21
Complication	(No. patients, %)	(No. patients, %)	
Blood transfusion	1 (1)	0 (0)	
Post void retention	2 (2)	7 (7)	
Stress Incontinence	9 (9)	2 (2)	
Urge Incontinence	7 (7)	4 (4)	
Urethral Strictures	2 (2)	1 (1)	
Bladder injury	1 (1)	1 (1)

**Table 3:** Postoperative functional outcomes (after 3 and 6 months)

	Group A	Group B	*P*
*3 months*			
Qmax ml/s (mean ± SD)	20.76 ± 9.78	22.42 ± 11.09	> 0.05
IPSS (mean ± SD)	6.12 ± 3.75	5.87 ± 5.18	> 0.05
PostVoid residual, ml (mean ± SD)	45.3 ± 25.16	42.3 ± 22.71	> 0.05
QOLS (mean ± SD)	44.2 ± 13.22	42.9 ± 11.86	> 0.05
*6 months*			
Qmax ml/s (mean ± SD)	19.43 ± 12.56	23.04 ± 8.54	> 0.05
IPSS (mean ± SD)	7.34 ± 5.43	5.45 ± 3.24	> 0.05
Post void residual, ml (mean ± SD)	31.9 ± 20.35	38.7 ± 21.62	> 0.05
QOLS (mean ± SD)	45.6 ± 11.59	41.8 ± 11.77	> 0.05

Complications in the two groups are presented in Table 2.

## Discussion

HoLEP is a surgical option for the management of BPH and an alternative treatment to TURP, or open prostatectomy, according to EAU Guidelines. One of the main advantages of HoLEP is that it reduces intraoperative and postoperative bleeding, leading to a lower transfusion rate, shorter hospitalization and catheterization [7].

This enucleating technique is performed with the Ho:YAG laser, which emits a pulsed laser beam, with a wavelength of about 2.1 µm, obtaining tissue vaporization, coagulation and necrosis limited to a depth of 0.3–0.4 mm [8]. The Ho:YAG laser is also used for stone lithotripsy, during which, the impact of the energy against the stone can cause its migration from the ureter to the renal cavities or from one calyx to another. To prevent this phenomenon, anti-retropulsion devices have been engineered, like the “Virtual Basket” mode, which is a result of the laser’s pulse modulation: the laser creates an initial bubble with the first part of its energy and discharges the remaining energy once the bubble is formed, so that it can pass through the formed vapor channel. In this study, we report our results on the application of the Virtual Basket mode to HoLEP (VB-HoLEP) compared to the conventional technique, with a 6-month follow-up.

Vizziello et al. firstly reported their in vitro experience regarding the use of the Virtual Basket in stone phantom lithotripsy [9]. The authors concluded that this mode was associated with significantly fewer events of stone migration and better target stability during the procedure. Another study [10] investigated this emission mode in the treatment of ureteral and renal stones. In particular, it was reported that when compared to the regular mode, the Virtual Basket technology was associated with significantly lower retropulsion, fragmentation time and total procedural time, with no significant differences in total emitted energy.

Based on these studies, this mode may grant a smoother effect not only on stones but also on soft tissues, resulting in less trauma and bleeding.

Because of its double pulse pattern, we hypothesized that the use of the Virtual Basket during HoLEP may result in a first energy portion creating an initial separation of the prostatic tissues and the remaining energy being discharged through the incision, expanding it further and clotting bleeding vessels. As the laser’s second pulse travels through the vapor tunnel created by the first pulse, a lower attenuation of the second pulse should occur, resulting in a stronger effect on the tissue (sealing and/or incision). This system, with the emission of two energy pulses fired with a small time gap between one another, seems to allow faster coagulation, reducing the risk of bleeding and, therefore, operative time. Indeed, as reported in our results, compared to HoLEP, the VB-HoLEP was faster (57.33 ± 29.71 vs. 42.99 ± 18.51 min, *P* = 0.04) and resulted in less hemoglobin decrease (2.54 vs. 1.12 g/dl, *P* = 0.003). Despite being inferior, compared to older surgical techniques, bleeding risk with HoLEP still remains. Some studies report a risk of severe hemorrhage in 5.2% of patients and a risk of bladder tamponade that required cystoscopy and evacuation of blood clots in 2.3% [11]. In some Centers, to reduce the risk of bleeding in the early post-operative period, surgeons use a bipolar resector to obtain prostatic loggia coagulation. This lengthens the operating time, increasing the risk of anesthesiologic complications. The use of the Virtual Basket could improve coagulation with the laser, allowing to avoid the use of the bipolar resector and reduce morcellation time, thanks to a good endoscopic vision, without residual bleeding.

Moreover, HoLEP has proven to be safe and effective in anticoagulated patients. The hemostatic efficacy of the Ho:YAG laser makes HoLEP more effective and safer than other BPH treatments in patients taking anticoagulant agents. Specifically, the low penetration depth of the holmium laser limits eschar formation, which can contribute to delayed bleeding seen after other BPH procedures [12]. The use of VB-HoLEP, thanks to its observed better coagulation capability, could further reduce the risk of bleeding in patients under anticoagulation therapy [13].

The comparison between HoLEP and VB-HoLEP during the 6-month follow-up did not demonstrate significant differences in Qmax, IPSS, PVR, and QOLS.

Urinary incontinence (UI) is one of the most worrying postoperative complications. Postoperative UI occurres in about 20 % of patients and most of them recover within the first year. Long operative time is the first risk factor: the longer the resectoscope remains in the urethra, the higher the possibility of sphincter damage. Some studies stated that high prostate volume, a conspicuous reduction in postoperative PSA and diabetes mellitus are significant risk factors for stress UI [14]. Various authors have suggested that postoperative incontinence could be related to either thermal injury to the pericapsular structures, resulting in urge incontinence, or linked to the presence of a urinary tract infection, or BPH-related detrusor instability [15]. Another risk factor for UI is the presence of a large prostatic fossa, created after the removal of adenoma, which leads to urine entrapment and leakage with stress maneuvers [16]. VB-HoLEP could reduce the risk of UI thanks to a better cut on the tissue, resulting in reduced traction and stress on the urethra and external sphincter.

The long learning curve is the major negative factor that hinders widespread use of this procedure to date [17]. The inexperience of the surgeon elevates the risk of bleeding and UI after HoLEP because of long operative time, frequent intraoperative complications and inadequate enucleation.

As the use of VB-HoLEP proved to reduce operative time in our study, the risk of UI may be reduced with this technique. Moreover, as the use of the Virtual Basket reduced bleeding, improving the quality of the endoscopic vision, the use of VB-HoLEP may help to reduce the learning curve. These aspects may be verified in future multicentric studies.

Together with the long learning curve associated with the enucleation technique, the cost associated with the purchase of high power laser platforms has probably represented another factor hindering the spread of laser enucleation. However, the possibility to use the VB technology, with reusable fibers and on medium power platforms might help to foster the adoption of HoLEP in upcoming years. Indeed, the non-inferiority of low-power HoLEP with respect to high power HoLEP has been investigated [18, 19]. For instance, Elshal et al. compared 50 W and 100 W HoLEP techniques, reaching comparable improvement in IPSS, Qmax and median PSA reduction, with similar perioperative and late postoperative complications [18].

There is growing interest for new pulse modulation technologies which can potentially enhance lithotripsy effectiveness and which have been recently launched on the market [9, 20, 21]. However, so far the potential advantages of these modulations have been mainly explored for stone application, whereas little has been reported regarding the effect of these pulse modulations on soft tissue treatments. One exception consists in the study performed by Large et al., who shared their experience with the Moses™ technology for HoLEP [22]; Large et al. reported that the use of this modality resulted in increased OR efficiency and hemostasis, regardless of prostate size, when compared to standard HoLEP. Both his study and ours suggest that advanced pulse modulation of the Ho:YAG laser results in increased hemostatic effect. Nevertheless, there are differences between these two technologies. First, the second pulse of Virtual Basket is emitted when the vapor bubble, originated by the first pulse, is at the maximum expansion; instead, the second pulse of the Moses is emitted during the collapse of the bubble originated by the first pulse. Moreover, another difference is in the fiber compatibility with these pulse modulations: Virtual Basket, unlike the Moses, is pulse modulation which is compatible with any standard fiber, without the need of a “special” fiber in order to enable this pulse modulation.

To our knowledge, this is the first study describing the use of the Virtual Basket mode for HoLEP and one of a small group of papers reporting the use of advanced Ho:YAG pulse modulation for soft tissue applications, such as the Moses™ technology, which has been used since 2017. Further investigations by other centers are needed in order to corroborate the findings of our study.

Limitations of this study are linked to the fact that all the procedures were not performed by only one skilled surgeon. Another limitation is that hemostasis effectiveness was judged only by measuring hemoglobin drop. Potentially, recording of the time spent on hemostasis (for example the time with the right pedal pushed) may have represented an additional comparison term to corroborate our outcomes regarding hemostatic properties. Furthermore, only a single emission setting was tested in this study for both groups.

## Conclusions

Compared to conventional HoLEP, VB-HoLEP determines faster operative time and results in less hemoglobin decrease, due to better coagulation, but there are no differences with regard to catheterization time, irrigation volume, hospital stay, Qmax, IPSS, PVR and QOLS at 3 and 6 months. Based on these results VB-HoLEP may be better than conventional HoLEP, but from our experience in the field of laser enucleation of prostate, it may not overcome the efficacy, safety and early and late outcomes of thulium laser enucleation of the prostate (ThuLEP) [23].

## Data Availability

the datasets used and/or analysed during the current study are available from the corresponding author on reasonable request.

## Abbreviations

BPH: Benign prostate hyperplasia
HoLEP: Holmium laser enucleation of the prostate
IPSS: International Prostate Symptom Score
LUTS: Lower urinary tract symptoms
OP: Open prostatectomy
PVR: Postvoid residual urine volume
Qmax: Maximum flow rate
VB: Virtual Basket
VB-HoLEP: Holmium laser enucleation of the prostate with Virtual Basket mode
